# Refractory post-thymectomy myasthenia gravis with onset at MGFA stage V: a case report

**DOI:** 10.1186/s13019-022-01872-0

**Published:** 2022-05-12

**Authors:** Rui-Qin Zhou, Lin-Jun Li, Qing-Chen Wu

**Affiliations:** Department of Cardiothoracic Surgery, The First Affiliated Hospital of Chongqing Medical University, Chongqing, 400016 China

**Keywords:** Post-thymectomy myasthenia gravis, Case report, Thymoma, Infection, Misdiagnose

## Abstract

**Background:**

Post-thymectomy myasthenia gravis (PTMG) is defined as thymoma patients without signs of myasthenia gravis (MG) pre-operation, but develop MG after radical surgical resection. PTMG might be misdiagnosed not only because of its rare incidence, but also the uncertain interval between the removal of thymoma and the new onset MG. Additionally, some surgeons and anesthesiologists pay less attention to those asymptomatic thymoma patients in perioperative management, leading to the neglect of new onset PTMG, and miss the best time to treat it.

**Case presentation:**

Majority of cases of PTMG with onset at stage I–II on the basis of Myasthenia Gravis Foundation of America (MGFA) classification have been reported, but rarely at stage V, which requiring intubation or non-invasive ventilation to avoid intubation. Herein, we presented a 70-year-old male with PTMG onset at MGFA stage V, meanwhile, he had severe pulmonary infection interfering with the diagnosis of PTMG, and eventually progressed to refractory PTMG, which requiring much more expensive treatments and longer hospital stays.

**Conclusion:**

In the perioperative management of asymptomatic thymoma patients, careful preoperative evaluation including physical examination, electrophysiological test and acetylcholine receptor antibodies (AChR-Ab) level should be done to identify subclinical MG. Complete resection should be performed during thymectomy, if not, additional postoperative adjuvant therapy is neccessary to avoid recurrence. It’s important to identify PTMG at a early stage, especially when being interfered with by postoperative complications, such as lung infection, so that treatments could be initiated as soon as possible to avoid developing to refractory PTMG.

## Background

Post-thymectomy myasthenia gravis (PTMG) is that thymoma patients who have no signs of MG before surgery, but develop myasthenia gravis (MG) after radical surgical resection. PTMG might be misdiagnosed not only because of its rare incidence, 0.97–13.39% in previous studies [1–8], but also the uncertain interval between the removal of thymoma and the new onset of PTMG, ranged from 3 days to over 14 years [3, 7]. There have been reported majority of cases of post-thymectomy MG with onset at stage I-II on the basis of Myasthenia Gravis Foundation of America (MGFA) classification, but rarely at stage V, which requiring intubation or non-invasive ventilation to avoid intubation [9]. We present the patient of PTMG with onset at MGFA stage V, meanwhile, he had severe lung infection, which interfering with the diagnosis, and unfortunately he eventually developed to refractory PTMG.

## Case report

A 70-year-old male was hospitalized with intermittent chest pain. He had no background diseases and his physical examination was unremarkable. The computed tomographic (CT) scan showed an anterosuperior mediastinal mass surrounded large blood vessels, and positron emission tomographic (PET) scan revealed no evidence of a metastatic tumor. The thoracic CT angiography showed the shape of the vessel wall was still regular, and there was no obvious sign of filling defect. Thymoma was the most likely diagnosis and could be directly surgically resected post discussion and evaluation by our multidisciplinary team. As the patient had no signs of MG at that time, anti-AChR antibodies test and electromyogram were not performed. His lung function was normal. In April 2020, he received a median sternotomy, thymothymectomy, pericardiectomy and left upper lobectomy were performed to ensure a complete resection. Histology of the tumor showed a thymoma type B2 (WHO classification), Masaoka stage III. 9 days later, he was discharged without any postoperative complications. However, 14 Days after the surgery, he complained of dyspnea and fever, thoracic CT showed no evidence of tumor reccurence, but increased patchy density was seen in both lower lungs. He had a sudden loss of consciousness in the emergency room, and the blood gas analysis showed a type II respiratory failure with the level of partial pressure of carbon dioxide over 100 mmHg, endotracheal intubation and ventilator assisted ventilation were performed immediately. Severe pneumonia was diagnosed according to the clinical outcome, confirmed by the result of bacterial culture of bronchoalveolar lavage fluid, acinetobacter baumannii. With sensitive antibiotics treatment, the lung infection was controlled and his condition was markedly improved. However, we failed to wean him off ventilation. Neostigmine test was negative in this patient, but repetitive nerve stimulation test was positive and the titer of anti-AChR antibody was slightly elevated, at 0.43 nmol/L (cut-off value for positive: > 0.5 nmol/L, questionable positive: 0.4–0.5 nmol/L, negative: < 0.4 nmol/L), the neurologist suggested that PTMG should be taken into consideration, as patients with MG often require prolonged ventilatory support, we did tracheostomy to facilitate his weaning of the ventilator on day 10 after endotracheal intubation. With the administration of pyridostigmine bromide (180 mg/d), he could gradually wean off the ventilator. Nevertheless, he suffered form refractory diarrhea, a serious side effect of pyridostigmine bromide, then we discontinued the medications, leading to his ventilation requirement again, therewith, he was referred to neurology care unit for specialist treatment. Intravenous immunoglobulin (IVIG) was suggested as an optimal treatment, but was refused for its high cost at first. Steroid pulse therapy was initiated instead, unfortunately the effect was slow and poor, so plasmapheresis and IVIG were administrated successively. At 4 months after his admission, he was eventually removed from the ventilator with a low dose tacrolimus of 1 mg/day.

## Discussion

Myasthenia gravis (MG) is caused by an autoimmune antibody against acetylcholine receptors on the neuromuscular junction, which usually coexists with thymoma. The incidence of thymoma in MG is about 10%-12%, and that of MG in thymoma is approximately 30% [4, 6, 10]. Complete resection is the gold standard for treating thymoma. The complete remission rate after thymectomy in preoperative MG patients was 44.1–59.5% according to previous studies [11–13]. However, asymptomatic thymoma patients can develop PTMG. The mechanism of the onset of PTMG is unclear. Risk factors include a positive result for serum AChR-Ab, incomplete resection, and postoperative infection [7].

Some studies supported that elevated level of preoperative AChR-Ab could be proof of an subclinical MG [5, 6, 8]. Ito et al. divided their patients with PTMG into early and late onset group respectively, and found the early onset group may have had subclinical MG when they underwent initial surgery [2]. Previous studies revealed the incidence of PTMG in preoperative AchR-Ab positive patients were 23, 18.75 and 30.3%, respectively, and those of PTMG in preoperative AchR-Ab negative patients were 4.76, 2.78 and 0%, respectively [5, 7, 8], which suggested that preoperative high level of AchR-Ab might be a predictive indicator for the development of PTMG. In our case, there was a limitation that the patient’s preoperative Ach-Ab serum level and electromyogram weren’t measured. Although the postoperative serum titer of AChR-Ab was just slightly elevated, the clinical symptoms of PTMG were quite serious, which indicated that AChR-Ab level had a poor correlation with the clinical severity of MG patient [14].

In the late onset group of Ito’s study, they found PTMG was associated with tumor recurrence [2]. Mineo et al. redid surgeries to remove residual fat tissue where increased metabolic activity were detected in the late onset PTMG patients, resulting in complete remission [8]. However, in our case, the patient wasn’t found any evidence of recurrence, Kondo et al. also didn’t find any amelioration in PTMG patients after the resection of the residual thymus gland, which indicated extrathymic mechanism may play a role in the production of AchR-Ab [4]. Studies found mature autoantigen-specific T cells coming from thymomas could persist in the peripheral blood for many years [9, 15].

In our case, we blamed the patient’s respiratory failure on the severe pneumonia initially, without awareness of coexisting PTMG. Actually, pneumonia is a common complication after thoracic surgeries, mainly attributed to the stagnation of airway secretion, which can be also caused by respiratory muscle weakness in MG patients. Infection can not only induce MG, but also lead to MG deterioration [16]. As seen in our case, his severe pneumonia may be the result of MG, which in turn leads to MG deterioration, forming a vicious cycle. Combined with our unwareness of new onset PTMG, the patient missed the best opportunity for treatment, and finally developed to refractory PTMG, requiring much more expensive treatments and longer hospital stays.

## Conclusion

In the perioperative management of asymptomatic thymoma patients, careful evaluation including physical examination, electrophysiological test and AChR-Ab level should be done to identify subclinical MG, so that the surgeons and anesthesiologists could take measures in advance to avoid PTMG. Complete resection should be performed during thymectomy, if not, additional postoperative adjuvant therapy is neccessary to avoid recurrence. It’s important to identify PTMG at a early stage, especially when being interfered with by postoperative complications, such as lung infection, so that treatments could be initiated as soon as possible to avoid developing to refractory PTMG.

## Data Availability

Not applicable.

## Abbreviations

PTMG: Post-thymectomy myasthenia gravis
MG: Myasthenia gravis
MGFA: Myasthenia Gravis Foundation of America
AChR-Ab: Acetylcholine receptor antibodies
CT: Computed tomography
PET: Positron emission tomography
anti-AChR: Anti-acetylcholine receptor
